# Empathic Accuracy in Clinical Populations

**DOI:** 10.3389/fpsyt.2020.00457

**Published:** 2020-06-03

**Authors:** Yonat Rum, Anat Perry

**Affiliations:** Psychology Department, The Hebrew University of Jerusalem, Jerusalem, Israel

**Keywords:** empathic accuracy, autism, schizophrenia, psychopathy, depression, anxiety, behavior disorders, personality disorders

## Abstract

Empathy, broadly defined as the ability to understand the other and to share others’ emotions, motivates prosocial behavior and underlies successful interpersonal relations. Dysfunctions in this ability may cause fundamental difficulties in social communication. Empathy has been measured in various ways, from self-report questionnaires to laboratory objective performance tests. Empathic accuracy (EA), i.e., the ability to accurately empathize, is measured using more complex and ecological paradigms, such as asking participants to infer filmed interactions, or having people narrate personal emotional stories then assessing the correspondence between the perceiver and the target of empathy as the criteria for empathic ability. This measure is particularly useful in the study of clinical populations, where deconstructing the multifaceted concept of empathy may contribute to a more complete understanding of specific clinical profiles. This paper presents a scoping review of the literature on EA in clinical populations, and on EA and clinical traits and states in nonclinical or high-risk populations. Following an exhaustive literature search, 34 studies were found eligible to be included in this review. The largest category was studies focused on EA in people with schizophrenia (31%; 11 papers), followed by studies focused on EA in autism spectrum disorders (ASD) and autistic traits in a nonclinical population (22%; 8 papers). Studies were also found on EA and depression tendencies, psychopathy, social anxiety, behavior disorders, and personality disorders, and a few other clinical conditions. The included studies varied on research aims, designs, sample sizes, and male:female ratios. The overall synthesized results suggest that EA is reduced in schizophrenia and ASD. In other clinical populations, the number of studies was very limited. We urge researchers to further examine EA in these less-studied populations. The review reveals a general underrepresentation of female participants in studies on EA in clinical populations. We suggest that future research address understudied clinical populations, such as those diagnosed with psychopathy. Subject, target, and situational variables should also be considered, with special attention to gender differences (and similarities), the association between EA abilities and adaptive functioning, and the study of individuals with clinical conditions as targets, not just observers, in EA tasks.

## Introduction

Every well-adjusted social interaction—for example, between parents and children, between peers or between partners—requires recognition, understanding and sometimes sharing each other’s thoughts, feelings, and emotions. Applying these complex skills, while maintaining a self/other distinction, is termed *empathy* (1–3). An evolutionary perspective suggests that the basic need to care for offspring explains why human beings developed empathy (4). Empathy motivates prosocial behavior and interpersonal relations (2). On the other hand, dysfunction or lack of empathic abilities may cause not only misunderstandings and unpleasantness but also fundamental difficulties living in society. The multifaceted concept of empathy can be divided into *cognitive empathy* (or *mentalizing*)—the recognition and understanding of others’ mental states—and *emotional empathy* (or *experience sharing*)—in which the affective experience is similar to that of the other, or there is an emotional response to the mental state of the other [(5–7); for a review see: (2, 8)].

As a sophisticated yet fundamental ability that plays a central role in human relationships, empathy has been extensively researched for decades, and it has been examined specifically in clinical populations in which social dysfunctions are key. For example, autism spectrum disorder (ASD), psychopathy and schizophrenia are clinical conditions that according to several theories are associated with pronounced empathic dysfunction (9–15). In both ASD and psychopathy, a social deficit is not only a characteristic, it is a diagnostic criterion (16, 17). While some have suggested that both cognitive and emotional domains of empathy are impaired in schizophrenia (18), others claim that schizophrenia and psychopathy are characterized by deficits in emotional empathy but not cognitive empathy (11, 19, 20). Aberrant empathic functioning, specifically impairments in cognitive empathy, was also found in borderline personality disorder [BPD; (21)] and bipolar disorder (22), two conditions associated with interpersonal deficits. However, other findings support a hypothesis according to which individuals with BPD are uncommonly sensitive or “over empathic” to the internal experience of others (23–25). Findings from clinical populations are of great value for understanding the multifaceted concept of empathy on the one hand, and specific clinical profiles on the other hand, but these are not always consistent. One possible explanation for the inconsistencies may be the varied operationalizations of empathy in research.

Researchers in the fields of developmental, social, cognitive, educational, and clinical psychology, as well as cognitive neuroscience, use different methods and instruments to measure empathy in the general population, and in clinical or high-risk populations. In early childhood, empathy is often measured through observations, as a behavioral response to a simulation of others’ distress (26–28) or by caregivers’ reports [e.g., (29)]. In older children, empathy is measured using different tasks, including the evoked emotional response in the child (30, 31). In schoolchildren, adolescents, and adults, empathy can be measured using either self-report questionnaires [e.g., IRI, (32); EQ, (33); CEAQ, (34)] or objective performance tests, which compare participants’ output to predefined “correct” responses. These kinds of tasks include emotion recognition tasks in still pictures, and reading a vignette describing a mental state or a social situation. Theory of Mind (ToM) and traditional false beliefs and “Faux-Pas” tasks are also related to some extent to the cognitive component of empathy (35–40). Generally, some tasks or questionnaires primarily measure the cognitive empathic component, while others capture more of the emotional output. Objective performance tests offer some integration between the individual’s perspective and the observed behavioral output, but such laboratory tasks usually fail to capture the dynamic nature and complexities involved in social communication, including rapid and nuanced changes in facial expression, intonation and other pragmatic characteristics of the speech, posture and gestures of the target social partner (41). Other limitations in some of the methods mentioned above include the fact that reading and comprehension abilities and executive functions (e.g., in questionnaires, vignettes) may present a potential confound, and the fact that some measures refer to a very narrow aspect of empathy (e.g., emotion recognition from facial expressions). In studying empathy in clinical populations, these limitations need to be considered.

Empathic accuracy (EA) tasks have tried to offer a more ecological setting to measure empathic abilities. EA is the ability to accurately judge the cognitive and affective mental states of others (42, 43). Accordingly, in the original lab procedure developed by Ickes and his colleagues, a dyad is videotaped while interacting. Then each member of the dyad views the videotape separately and reports his or her own thoughts and feelings during the interaction, as well as inferences regarding the partner’s thoughts and feelings during the interaction. EA is measured by the similarity between the explicit reported mental states of the target and those reported by the perceiver (42, 43). In the current review we refer to this prototype paradigm (and later adaptations and variations of it) as a dyadic interaction paradigm.

A more recent EA paradigm developed by Zaki and colleagues is based on the perceiver’s interpretation of a target’s videotaped autobiographical emotional story as the stimulus (instead of a dyadic interaction), and the correspondence between the perceiver’s and the target’s ratings of *valence* (i.e., how positive or negative the target felt while telling the story) instead of the exact mental content. In this paradigm, both the target and the perceiver use a rating dial to continuously rate the valence of the videotaped story, and the perceiver’s EA score results from the correlation between the two continuous ratings (41, 44, 45). This method comes from an earlier attempt by Levenson and Ruef (46) to create a measure of behavioral empathy that relies on rating dials to provide continuous responses to a given videotaped stimulus. Here, we refer to this method as an emotional story inferring paradigm.

Another EA paradigm, which has been utilized mostly in research on romantic partners, uses experience-sampling diaries [e.g., (47, 48)]. In this approach, participants provide daily reports of their own mental states and their inferences regarding their partner’s perceived mental states over a period of time. Then reports of each participant on his/her partner’s (the target’s) thoughts and feelings are compared to the target’s own reports to arrive at an EA score. In this review, this is referred to as a daily diary paradigm.

All three prototypes of EA paradigms yielded various studies, and some of them applied specific variations and adaptations to the original developed tests. Common to all is the reliance on the concordance between the perceiver’s (the subject of the EA measure) view of the target and the target’s (the object of the EA measure) own report on their internal states to generate the EA measure. As such, EA measures provide more ecologically valid data on interpersonal perception in comparison to other experimental techniques. Moreover, an fMRI study by Zaki and colleagues suggests that both cognitive and emotional mechanisms contribute to the ability of the perceiver to accurately match her state with the emotions or thoughts experienced by a social target (49). Thus, measuring EA seems to capture a more nuanced measure of empathy and reflect its complexity.

The main objective of this review is to provide an overview of the existing literature on EA in clinical populations or high-risk subclinical populations, and on clinical states and traits measured in nonclinical samples. To this end, we aim to (1) conduct a systematic search of the published peer-reviewed papers on EA in clinical populations; (2) map the characteristics and range of findings and conclusions in the identified papers; (3) examine reported challenges and limitations of measuring EA in clinical populations; and (4) propose recommendations for future research directions. Within the scope of this review are studies measuring EA conducted on clinical populations, as well as studies focusing on clinical traits in a high-risk or nonclinical population. We considered studies measuring valence (negative-positive) or content (thoughts, feelings), and measuring EA as a primary or secondary aim of the study (for example, studies measuring EA in a clinical sample as part of a battery of tests assessing social cognition). We also considered a variety of paradigms used to assess EA, including the dyadic interaction paradigm, the emotional story inferring paradigm and the daily diary paradigm. Common to all studies was the aim to assess the perceivers’ ability to accurately understand and report on the targets’ affective or mental state when the criteria are the target’s own representations of his or her mental state.

## Methods

The methodology was based on the framework outlined by Arksey and O’Malley’s (50) review and recommendations made by Levac et al. (51). It consisted of five key phases: (1) identifying the research question; (2) identifying potentially relevant studies; (3) selection of studies; (4) charting the data; and (5) organizing, summarizing, and reporting the findings. The Preferred Reporting Items for Systematic Reviews and Meta-Analyses (52) were used in the current review as a guide, where applicable.

### Research Question

This review was guided by the following two questions: (1) What are the characteristics of studies measuring EA in clinical populations? and (2) What are the main findings and conclusions in the literature regarding EA in clinical populations? For the purposes of this review, all papers that used the term “empathic accuracy” and referred to a concordance or correlation between two partners (i.e., a target and a perceiver) were included.

### Data Sources and Search Strategy

The initial search was implemented in July 2019, using PsycNET and PubMed. The search query included the term “empathic accuracy” AND (permutations of) the terms: “autism”; “psychopathy”; “schizophrenia”; “depression”; “dyslexia”; “attention deficit”; “anxiety”; “OCD”; “behavior disorders”; “personality disorders”; “mood disorders”; “affective disorders”; “neurodegenerative disease”; “mental disability”; “learning disability”; “neurodevelopmental disorder”; “clinical population”; “mental disorders.” The reference lists of all potentially relevant papers were screened in a two-phase process: (a) title and abstract screening; and (b) full-text screening. Empathy measures were examined in the selected studies with respect to the extent to which they tapped into EA. A “snowball” technique was also utilized in which citations within papers were searched to look for potentially relevant studies. A follow-up search was conducted on September 24, 2019, to identify any additional relevant papers published after the initial search, resulting in the final list of papers for the review (see **Table 1**).

**Table 1:** Summary of studies’ characteristics.

Study	Objective/Research Question	Design and Participants	EA paradigm	Main conclusions regarding EA
**Schizophrenia Spectrum and Psychotic Disorders**				
van Donkersgoed et al. (53)	To assess the moderating role of the target’s gender and expressivity and the valence of the story on EA performance; the correlation between EA and other commonly used empathy measures.	Schizophrenia group (n=92, 67 males) Nonclinical control group (n=42, 32 males) matched for age, gender, and education	Emotional story	Schizophrenia group performed worse than controls in EA. Individuals with schizophrenia benefit less from the emotional expressivity of targets. No correlations were found between EA and questionnaire scores, suggesting a distinction between self-report empathy and actual empathy performance.
de Jong et al. (54)	To investigate which measures of social cognition and metacognition are related to violent history in patients with psychotic disorder; which domains of metacognition were indicative of a violent history in psychosis.	Violent psychotic disorder in care at a forensic clinic for a violent crime (n=23) Clinical group 2: nonviolent psychotic disorder (n=27, all males) Nonclinical control group (n=33, all males)	Emotional story	EA differentiated between the violent and nonviolent psychotic patients, while scores on social cognition (such as ToM) and a metacognition scale did not. EA may offer an important contribution to statistical models of violence risk in psychotic disorder.
Harenski et al. (55)	To explore the hypothesis that lower EA and smaller brain volumes in regions implicated in social cognition are related to past suicide attempts in offenders with a psychotic disorder.	Criminals with a psychotic disorder and a history of suicide attempts (n=18, all males) Criminals with a psychotic disorder and no past suicide attempts (n=25, all males) Nonclinical group: criminals with no history of a psychotic disorder (n=59, all males) Nonclinical control group (n=26; all males)	Emotional story	Criminal offenders with psychotic disorders and suicide attempts had lower EA and smaller temporal pole volumes compared to the other groups. EA and temporal pole volumes were significantly associated with past suicide attempts independent of other risk factors.
Horan et al. (56)	To evaluate correlations of the Questionnaire of Cognitive and Affective Empathy (QCAE) in schizophrenia with EA (and other empathy measures).	Schizophrenia group (n=145, 108 males) Nonclinical control group (n=45, 32 males)	Emotional story	No significant association was found between the QCAE and EA performance in either group, indicating that self-reported beliefs about empathic characteristics are not necessarily correlated with an actual understanding of others’ affective states.
Davis et al. (57)	To assess whether oxytocin (OT) enhances the effectiveness of a social cognitive training. The final four sessions of training focused on improving EA.	Individuals with schizophrenia (n=27, all males) were randomly assigned to an OT condition (n=13) or to a placebo condition (n=14). (Double-blind drug administration with before and after treatment comparison)	Emotional story	Administration of OT before a psychosocial intervention targeting social cognition improved EA and not other measures of social cognition, in individuals with schizophrenia.
Ripoll et al. (58)	To test schizotypal personality disorder (SPD) participants and healthy controls on the EA paradigm and the Reading of the Mind in the Eyes Test (RMET).	SPD group (n=19, 13 males, 6 females) Nonclinical control group (n=19, 6 males, 13 females)	Emotional story	SPD individuals demonstrated lower EA than controls during negative-valence videos, associated with lower social support. RMET did not differ between groups, suggesting that EA paradigms may be more effective at capturing interpersonal dysfunction than static image tasks. Schizotypal severity, trait empathy and cognitive dysfunction did not account for the empathic dysfunction.
Olbert et al. (59)	To examine the relationship between EA (and three other social cognitive paradigms adapted from social neuroscience) and functionally meaningful outcomes in schizophrenia (incremental, external validity).	Within-subject design on participants with schizophrenia (n=173, 124 males)	Emotional story	The EA paradigm was found to have the broadest external validity, and it is the most recommended measure from the four paradigms that were evaluated. EA had a significant association with functional outcome measures: Higher EA was associated with greater nonsocial cognitive ability, functional capacity, social skills and community functioning.
Kern et al. (60)	To evaluate psychometric properties of EA (and three other social cognitive paradigms adapted from social neuroscience) to inform possible use in clinical trials that assess treatment-related changes in social cognition in schizophrenia.	Schizophrenia group (n=173, 124 males) Nonclinical control group (n=88, 57 males) within subject (test-retest) in the schizophrenia group	Emotional story	The EA task had the best psychometric properties of the four paradigms checked: The largest between-group difference was seen on EA; of all measures, only a long version of the EA task met acceptable test-retest reliability standards; EA task was the strongest measure in regard to practice effects.
Harvey et al. (61)	To examine the neural correlates of EA and targets’ expressivity in schizophrenia.	Schizophrenia group (n=15, 13 males) Nonclinical control group (n=15, 13 males)	Emotional story	Schizophrenia patients demonstrated impaired EA, failed to benefit from targets’ emotional expressivity (wherein controls did benefit from targets’ expressivity), and demonstrated reduced neural sensitivity to targets’ affective cues.
Lee et al. (62)	To determine the relative extent of impairment in social and nonsocial cognitive domains in schizophrenia and bipolar disorder patients compared with healthy controls.	Schizophrenia group (n=38, 21 males) Bipolar disorder group (68, 38 males) Nonclinical control group (n=36, 20 males)	Emotional story	Schizophrenia patients performed significantly worse on EA than bipolar patients and controls, who did not differ from each other. *see findings regarding bipolar patients under Bipolar Disorder*
Lee et al. (63)	To examine whether schizophrenia patients showed lower EA compared with controls; whether emotional expressivity of a target moderated group differences; whether EA is associated with self-reported trait empathy or clinical characteristics in the schizophrenia sample.	Schizophrenia group (n=30, 25 males) Nonclinical control group (n=22, 17 males)	Emotional story	Schizophrenia patients were impaired in EA relative to controls. Both groups showed better accuracy for positive- vs. negative-valence videos. Both groups showed greater EA for highly expressive targets, but this effect was significantly smaller in schizophrenia patients. EA was not related to the participants’ self-reports or clinical symptoms.
**ASD and Autistic Traits**				
Adler et al. (64)	To compare levels of empathic embarrassment accuracy among individuals with ASD with those of matched controls.	ASD group (n=17, 16 males, high functioning/Asperger’s syndrome) Nonclinical control group matched for age and IQ (n=24, 21 males)	A paradigm designed to measure empathic *embarrassment* accuracy^1^	The ASD group displayed less empathic embarrassment accuracy compared with the control group. Higher AQ scores predicted low EA in the ASD group (a marginal correlation).
aan het Rot and Hogenelst (65)	To investigate the influence of autistic traits and trait affective empathy on EA.	Nonclinical sample (n=100, 50 male and 50 female)	Emotional story	Perceivers with more autistic traits demonstrated worse EA, particularly when their trait affective empathy was relatively low. Higher perceiver EA was predicted by a higher perceiver affective empathy and the target being female (rather than male), but there was no significant interaction between these two predictors.
Demurie et al. (66)	To investigate and compare the mind-reading abilities of adolescents with ASD, adolescents with ADHD and typically developed (TD) adolescents.	ASD group (n=13, 12 males) ADHD group (n=13, 12 males) Nonclinical control group (n=18, 14 males) adolescents	Dyadic interaction In each dyad one of two targets was TD, and the other was ASD or ADHD	Adolescents with ASD demonstrated impairment on both EA and a static task. *see findings regarding ADHD under ADHD*
Bartz et al. (67)	To test whether normal variance in social proficiency moderates the effects of oxytocin (OT) on social-cognitive performance.	Nonclinical sample (n=27, all males). Participants were randomly assigned to either an OT condition or a placebo condition, followed by an EA task. Participants returned 3 to 5 weeks later, received the alternate compound, and completed the EA task again.	Emotional story	Oxytocin selectively improved EA for people with higher (but not lower) autistic traits.
Ponnet et al. (68)	To investigate EA of participants with ASD asked to infer the mental states of targets in a highly structured conversation vs. a less structured/more naturalistic conversation.	ASD group (n=22, all males) Nonclinical control group (n=22, all males) matched for chronological age and IQ	Dyadic interaction One interaction was more structured than the other.	Differences between ASD and control groups in EA were more pronounced when participants had to infer the thoughts and feelings of other persons in a less structured conversation.
Ponnet et al. (69)	To measure the social functioning of adults with pervasive developmental disorder (PDD) during a conversation with a TD stranger and to explore whether EA of both groups was affected by behavioral characteristics and by the content of the interaction.	Part 1: Eleven dyads, each composed of a partner with ASD (n=11, 9 males; PDD) and a TD partner (n=11, 9 males), interacted in a lab task, then performed the EA task on each other within each dyad. PDD participants with the highest scores in the EA task of Roeyers et al. (70) were invited to participate in this study. TD participants were matched based on sex, age, education and main interests. Part 2: TD participants (n=13, 8 males), with the filmed interactions from part 1 as the stimuli for EA measure.	Dyadic interaction ASD participants take part in the interaction	No significant difference was found between controls and PDD participants in EA. No significant associations were found between EA and IQ scores, age or the time needed to complete the task. EA scores of the 11 participants with PDD correlated significantly with their EA scores on the previous study (Roeyers et al., (70); on a video of structured interaction). No significant difference was found among participants in part 2 in EA towards TD or PDD individuals as targets. Being in the interaction yields higher EA scores than just perceiving the interaction: participants in part 1 (PDD and TD) scored higher in EA than participants in part 2 (TD), who inferred EA from an interaction in which they did not previously take part.
Ponnet et al. (71)	To compare individuals with Asperger syndrome and controls’ performance in two static mind-reading tasks and the EA task.	ASD group (n=19, 14 males; Asperger’s syndrome) Nonclinical control group (n=19, 14 males)	Dyadic interaction	The EA task indicated significant between-group differences, whereas no such differences were found on the static mind-reading tasks. EA in both groups depended on the focus of the target’s thoughts and feelings. Participants with ASD needed more time than the controls to complete the EA task.
Roeyers et al. (70)	To compare individuals with PDD with controls on twopreviously used static empathy tests and onan EA task.	ASD group (n=24, 22 males; PDD/high-functioning) Nonclinical control group (n=24, 22 males) matched for sex, education, profession or interests	Dyadic interaction	Participants with PDD demonstrated worse EA in a video presenting a less structured conversation between two stranger targets, whereas no between-group differences were found in a video presenting a more structured conversation. Participants with PDD did not use more time than controls to complete the EA task. EA measure was proven to be a valid alternative to the previously used static tests.
**Depression Measured in a Nonclinical or High-Risk Population**				
aan het Rot et al. (72)	To examine the impact of light therapy on mood and on cognitive empathy in premenstrual women with complaints indicating a premenstrual disorder.	A nonclinical sample (n=48, all females) divided into two treatment groups (light therapy/sham session; participant-blind between-groups design)	Emotional story	There were no significant effects of light therapy on EA. Participants obtained higher EA scores when watching positive clips compared to negative clips.
Hogenelst et al. (73)	To investigate the effect of acute tryptophan depletion (ATD), which reduces brain serotonin, on social functioning, EA, and oxytocin levels.	High risk for MDD group (n=20, 10 males) Nonclinical matched control group A randomized, double-blind, crossover design (2 treatment conditions) with between-group comparison	Emotional story	EA remains unaffected by acute reductions in brain serotonin, even though brain oxytocin levels may be reduced.
Gadassi et al. (74)	To examine associations between EA and depression as a possible mechanism underlying gender differences in the association between interpersonal difficulties and depression in an intimate relationship.	Nonclinical sample of romantic couples (51 dyads; measurement of subclinical depression traits in couples)	Dyadic interaction and Diary	Depressive symptoms were associated with lower EA among females and may have a stronger impact on interpersonal perception in intimate relationships among females than among males. When a female is depressed, both her own and her partner’s EA levels are lower. When males are depressed, neither their own nor their partner’s levels of EA are lower. Depressive symptoms predicted lower EA regarding negative moods and feelings, but not regarding positive ones.
Papp et al. (75)	To examine affectivity in marital interaction: to test partners’ EA and assumed similarity in marital conflict interactions and whether they are moderated by spouses’ levels of depressive symptoms; to examine whether spouses’ ratings of their partner’s specific emotions depend on how they felt themselves in the same conflict interaction.	Nonclinical sample of romantic couples (267 dyads; measurement of subclinical depression traits in couples)	Dyadic interaction The interaction was focused on a topic of conflict	Females with higher levels of depressive symptoms demonstrated higher EA (and lower assumed similarity) compared to females with lower levels of symptoms. In rating negative emotions, spousal depressive symptoms weakened females’ abilities to rate their partners’ emotions; in rating positivity, higher females’ depressive symptoms strengthened their ratings of their partner’s emotions. Females’ depressive symptoms were associated with lower EA ratings *by their partners* (for anger, but not for sadness); males’ depressive symptoms were associated with lower EA in rating their partner’s anger. Males’ EA in rating their partner’s sadness was higher when their partner had a higher level of depressive symptoms. Partners of spouses with elevated depressive symptoms demonstrated particular difficulty in assessing partner anger in marital conflict.
Thomas et al. (76)	To examine the correlates of online EA in a sample of married couples in the context of problem-solving discussions, considering depression, relationship length and educational attainment.	Nonclinical sample of romantic couples (74 dyads; measurement of subclinical depression traits in married couples)	Dyadic interaction The interaction was focused on a topic of conflict	EA was not significantly correlated with depression in either males or females.
**SAD and Trait/State Social Anxiety**				
Morrison et al. (77)	To compare cognitive empathy and affective empathy in individuals with SAD to that of matched controls; to assess empathy with an adapted version of the EA task, with an additional behavioral index of *affective empathy*—by examining the degree of congruency between the target’s self-rating of emotion and the participant’s self-rating of his/her own emotions.	SAD group (n=32, 18 males) Nonclinical matched control group (n=32, 18 males)	Emotional story	No between-group differences were found in EA, indicating intact cognitive empathy in SAD. For positively valenced (but not for negatively valenced) clips, individuals with SAD exhibited significantly lower empathic congruence (affective empathy) than controls, indicating that affective empathy may be impaired in SAD.
Auyeung and Alden (78)	To examine whether individual differences in social anxiety moderate EA.	A nonclinical sample (n=121, 95 females) measured to assess social interaction anxiety in to conditions: experimental condition (a manipulation designed to increase state anxiety) and a control condition	Emotional story Specifically, targets narrated experiences when they felt: (1) socially excluded (2) socially included	Social anxiety was associated with greater EA for others’ social pain, but only when participants experienced social threat: Individuals with lower levels of social anxiety were less accurate in judging others’ negative emotions following a social threat.
Simpson et al. (79)	To test how people with more anxious-ambivalent attachment orientations react when their relationships are threatened by alternative dating partners.	Nonclinical sample of romantic couples (82 dyads; measurement of subclinical anxiety traits)	Dyadic interaction	Highly anxious-ambivalent individuals demonstrated higher EA (than those rated lower on anxiety) in a relationship-threatening situation (watching their partners rating opposite-sex optional dating partners), greater distress, and less confidence in their partners and relationships. The more anxious-ambivalent females reported a slight decrease in the perceived closeness of their relationships. More anxious-ambivalent males’ relationships were more likely to have ended by follow-up.
**Borderline Personality Disorder (BPD)**				
Miano et al. (80)	To investigate whether BPD patients show motivated inaccuracy by measuring their EA during a relationship-threatening conversation with their own romantic partner.	Dyadic analysis of BPD couples (30 couples; the female partner diagnosed with BPD) vs. a nonclinical control group of couples (34 couples)	Dyadic interaction Specifically focused on: (1) a personally threatening topic (2) a relationship-threatening topic	Reduced EA when facing a relationship-threatening situation was found in couples in the nonclinical control group, while females with BPD did not show this pattern of motivated inaccuracy and instead increased their EA, a finding that supports the concept of borderline empathy. Male partners of BPD females did not have a different EA pattern than control males. Neutral and personally threatening contexts did not significantly affect EA between BPD and control females.
Flury et al. (81)	To explore the phenomenon of borderline empathy (elevated empathy among individuals with BPD) with the use of EA.	A nonclinical sample (n=76, 46 females), composed of high vs. low risk for BPD, assigned to dyads each composed of a high-risk for BPD partner and low-risk for BPD partner.	Dyadic interaction In each dyad one “borderline” (high-risk) and one “nonborderline” (low-risk)	The empathic advantage displayed by high BPD individuals may not reflect greater ability, but result from the comparison to the ratings of their partner, who had difficulty inferring emotions of the BPD partners.
**Conduct Disorder and Callous-Unemotional Traits**				
Martin-Key et al. (82)	To assess EA, emotion recognition and affective empathy in male adolescents with Conduct Disorder (CD) and higher versus lower levels of callous-unemotional (CU) traits.	Clinical group: CD (n=37, all males) Nonclinical control group (n=40, all males) adolescents	Emotional story	Adolescents with CD did not differ in EA from TD adolescents but displayed significant impairments in emotion recognition and affective empathy (measured by asking participants to report whether they experienced the same emotion as the target). No difference in EA was found between high and low CU traits subgroups.
De Ridder et al. (83)	To assess everyday EA in institutionalized adolescents with high and low CU traits, and how EA is related to adolescents’ own behavior, and own affective and relational experience.	A sample of institutionalized adolescents (n=71, 45 males) divided into high CU traits vs. low CU traits *adolescents	A procedure similar to the diary paradigm^2^	High CU adolescents unexpectedly did not differ from low CU adolescents in EA (specifically inferring anger and distress in staff members) and notably overestimated the general intensity of both anger and distress, and in particular, inferred more anger when they (the adolescent themselves) were misbehaving.
**Psychopathy**				
Brook and Kosson (84)	To examine relationships between psychopathy and cognitive empathy. To design an improved EA task, with multiple targets, and a standardized forced-choice response format.	A sample of incarcerated offenders (n=103, all males)	Emotional story	Inverse association between psychopathy and EA was found, as well as robust group differences between psychopathic and nonpsychopathic inmates, findings that corroborate the deficient empathy hypothesis.
**Bipolar Disorder**				
Lee et al. (62)	See the same study in the category: Schizophrenia spectrum and Psychotic disorders.			Bipolar groups did not differ from the control group on EA but outperformed the schizophrenia group. Bipolar patients performed significantly better on social relative to nonsocial cognitive domains, whereas schizophrenia patients showed the opposite pattern.
**Risk for Hypomania**				
Devlin et al. (85)	To utilize a naturalistic, dynamic social stimulus (EA paradigm) in order to investigate the relationship between hypomania risk and empathy.	Nonclinical sample (n=121, 69 females), divided into high vs. low risk for hypomania	Emotional story	Risk for hypomania was associated with elevated EA of increases in positive emotion for targets describing positive events; however, it was also associated with overestimating global positive emotion for targets describing negative events.
**ADHD**				
Demurie et al. (66)	See the same study in the category: ASD and autistic traits in a subclinical population.			ADHD did not significantly differ in EA from either the control group or ASD group; thus, it was determined to be an intermediate group between the clinical and nonclinical groups. Thoughts and feelings of target persons with ADHD seemed to be less easy to read than the thoughts and feelings of TD targets.
**Neurodegenerative Disease**				
Brown et al. (86)	To investigate whether deficits in EA in patients with neurodegenerative disease are associated with greater depression in their caregivers.	Two independent cross-sectional samples (n=172, n=63) of patients with a variety of neurodegenerative diseases and their caregivers (usually spouses) vs. a nonclinical control group of healthy couples.	Dyadic interaction	Lower EA in patients was associated with higher depression in their caregivers. In study 1, this relationship was found using EA (after controlling for patient cognitive and functional symptoms) and was not found when using other more traditional tasks. In study 2, the relationship was found after accounting for caregiver characteristics that have previously been associated with caregiver depression.

^1^Participants watch films in which protagonists performed embarrassing actions and are asked to rate how embarrassed they feel (empathic embarrassment-EE) and how embarrassed they think the protagonist feels. The participant’s ratings are compared with the protagonist’s own ratings to produce a measure of empathic embarrassment accuracy.

^2^Adolescents reported the intensity of anger and distress they perceived in staff members; staff members reported their own levels of anger and distress after each period of at least 1 hour spent with the adolescent.

### Eligibility Criteria

Peer-reviewed papers written in English were eligible for inclusion if they explicitly aimed to measure *“empathic accuracy”* (EA) in a clinical population. Studies published in any year were considered. Papers that referred to *empathy* in clinical populations without measuring EA and review papers were excluded from the analysis, but their reference list was reviewed to identify additional relevant papers. Papers aiming to measure the relation between EA and *clinical traits* in nonclinical populations were also included in the current review. Exclusion criteria included: papers in languages other than English; nonpeer-reviewed papers (such as theses or dissertations); and papers aiming to measure EA in the general/nonclinical/typically developing population.

### Data Characterization and Analysis

All papers deemed relevant after the title and abstract screening were procured for subsequent review of the full text. Studies were excluded at this phase if they were found not to meet the eligibility criteria. The following characteristics of each full-text article were then extracted: objectives; participants (clinical population, N, age, gender); definition of EA; EA paradigm used; main findings and main conclusions regarding EA. All references, abstracts and data characteristics were imported into Microsoft Excel. Descriptive statistics were calculated to summarize data characteristics when applicable. The main findings and conclusions of all reviewed papers were discussed in light of the known data characteristics, limitations, and strengths of the included studies.

## Results

### Search and Selection of Papers

The original search conducted in July 2019 yielded 17 potentially relevant citations for EA and *“autism”* (using “ASD” as a search word instead of “autism” yielded no additional papers). For EA and *“schizophrenia,”* 24 potentially relevant citations were found (using “schizophrenic” as a search word instead of schizophrenia yielded no additional papers). For EA and *“psychopathy,”* four potentially relevant citations were found. For EA and *“depression,”* 26 potentially relevant citations were found (using “depressive” as a search word instead of “depression” yielded one additional potentially relevant paper). For EA and *“attention deficit,”* five potentially relevant citations were found (using “ADHD” as a search word instead of “attention deficit” yielded no additional papers). For EA and *“anxiety,”* 22 potentially relevant citations were found. For EA and *“behavior disorders,”* 16 potentially relevant citations were found (using “conduct disorder” or “disruptive behavior disorders” as a search word instead of “behavior disorders” yielded one additional potentially relevant paper). For EA and *“personality disorders,”* five potentially relevant citations were found (using “borderline disorder” as a search word instead of “personality disorders” yielded no additional papers). For EA and *“neurodegenerative,”* one potentially relevant citation was found (using “degenerative” or “Alzheimer’s disease” or “Alzheimer” or “dementia” as a search word instead of “neurodegenerative” yielded no additional papers). One potentially relevant paper was found for EA and *“learning disabilities”* (using “learning disability” as a search word instead of “learning disabilities” yielded no additional papers). No potentially relevant papers were found for EA and *“dyslexia”* or “dyslexic”, for EA and “*OCD*,” for EA and *“mood disorders,”* or for EA and *“epilepsy*.” No potentially relevant papers were found for EA and *“mental disabilities”* or *“mental disability,”* or for EA and *“clinical populations.”* For EA and *“mental disorders,”* five potentially relevant citations were found in the search.

**Figure 1:**
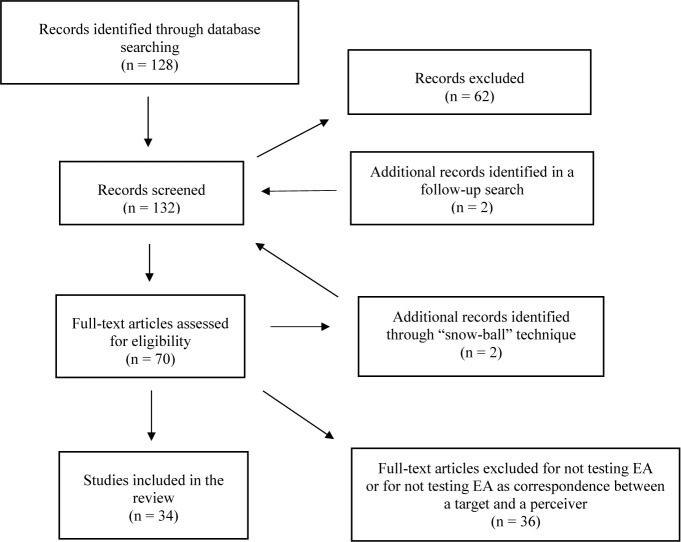
Studies search flow diagram.

Thus, the initial list consisted of 128 references. After the first phase of relevance screening, 70 citations were considered to potentially meet the eligibility criteria based on title and abstract, and the full-text articles were reviewed. In the second phase of reviewing full texts, 34 papers were excluded. Among the excluded papers, two mentioned measuring EA, but no results regarding EA were reported, and two papers were not available. During the full-text screening, the “snowball” search technique resulted in two additional eligible papers. The updated search in September 2019 produced two more potentially relevant citations, one of which was found to be eligible and was included. During the full-text screening phase, two studies were excluded, as the current inclusion criteria referred to EA as a measure comparing the subject’s perception to the target’s own perceptions: one study (87) that used the term “empathic accuracy” to refer to the “Reading the Mind in the Eyes” test [RMET; (36)], and one (88) that referred to EA as the correlation between a perceiver’s rating and a panel of judges’ ratings of the emotions of the same target (and not the concordance between the perceiver’s and the target’s rating). Thus, the final list of papers selected for inclusion in the current review consists of 34 peer-reviewed papers. **Figure 1** presents the search flow diagram.

### Characteristics of Included Papers

#### EA Definition

An explicit definition or description of what the authors mean by *“empathic accuracy”* was reported in all but four of the papers. Most definitions/paradigms centered on *the ability to accurately judge the valence and/or content of emotions or thoughts experienced by another person*, mostly citing Ickes et al. (43), Ickes (42), and Zaki et al. (44). However, there was some divergence in how authors characterized EA. Some studies referred to EA as a measure of cognitive empathy (53, 65, 73, 77, 83, 84). In contrast, some other authors mentioned that because EA is the ability to correctly infer the *emotional* state of a target, it has a relatively affective character (54, 75). Harvey et al. (61) claim that EA is not solely a measure of mental-state attribution (associated with cognitive empathy) or of experience sharing (associated with affective empathy), but that it is *the product of these two processes* [this definition was also used by Martin-Key et al. (82)].

#### Clinical Populations

Included papers referred to EA in the following categories of clinical populations and traits in high-risk, subclinical or nonclinical populations: schizophrenia spectrum and psychotic disorders (31%; 11 papers); ASD and autistic traits in a nonclinical population (22%; eight papers); depression measured in a nonclinical or high-risk population (14%; five papers); social anxiety disorder (SAD), social anxiety, and trait/state anxiety in a nonclinical population (8%; three papers); BPD (5%; two papers); conduct disorder and callous-unemotional traits (5%; two papers); and one paper in each of the following categories: psychopathy; hypomania; attention deficit and hyperactivity disorder (ADHD); bipolar disorder and neurodegenerative disease. Two papers were assigned to two categories, as they compared two clinical samples in the study [an ASD group was compared to an ADHD group in Demurie et al. (66); a bipolar disorder group was compared to a schizophrenia group in Lee et al. (62)]. In most of the papers, learning about the nature of EA in a clinical population was the primary aim; thus, a between-group design was assigned, comparing the clinical group to a matched control group. In some of the studies, this was a secondary aim, as when EA was part of a battery of tests to assess social cognition (62), or when the primary aim was evaluating interventions (57, 67, 72, 73) or evaluating the psychometric properties of an EA paradigm (59, 60).

#### Clinical Sample Sizes

Of the final list of eligible papers, 23 (67%) reported studies done directly on participants from a clinical population (i.e., participants have a diagnosis of one of the above-mentioned conditions), while the rest referred to clinical traits in healthy, nonclinical or high-risk populations. Of the 23 studies that included participants with a clinical diagnosis, the largest sample size was n = 173 [(59, 60)]; schizophrenia spectrum and psychotic disorders category), and the smallest sample size was n = 11 [(69)]; ASD category), with 48% (11/23) of the studies based on n < 30. In the studies with nonclinical or high-risk populations, samples were usually larger, with all studies but one ((67); n = 27) based on n > 30, and 6 of them with sample size of n > 100 (see **Table 1**).

#### Male : Female Ratio

**Figure 2:**
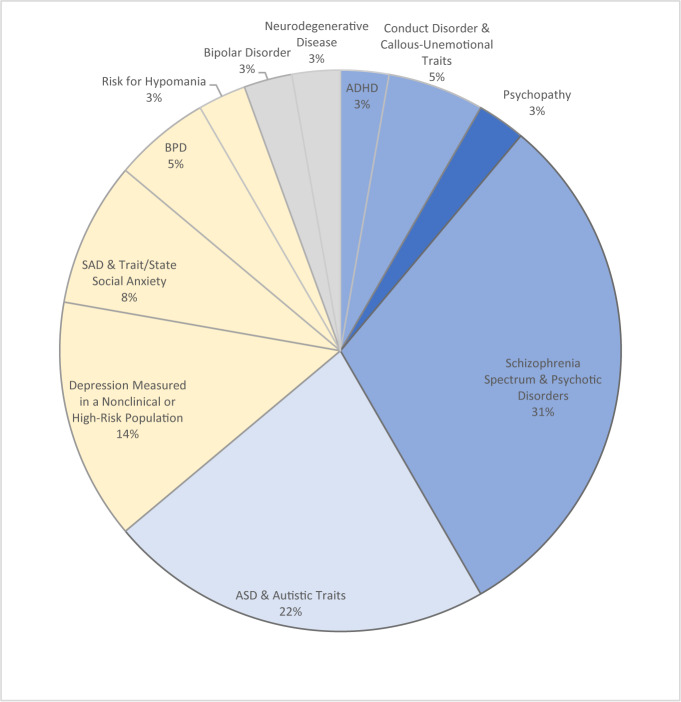
Distribution of studies by clinical population category. Percentages refer to the percentage of papers on that population out of all papers in the current review, and colors refer to the male:female ratio. In blue, categories with overall more males than females among all participants (in all studies together). In yellow, categories with overall more females than males among all participants. The darker the color, the more pronounced the underrepresentation for females, with darker blue = 0 females, lighter blue = a ratio of more than 3:1, lightest blue = a ratio of more than 2:1, and gray = a ratio of less than 2:1. Specific male:female ratios for each category are reported under “Specific Results per Clinical Population”.

In six studies where EA was measured on clinical samples, there was no representation of females (0 female participants; see **Table 1**). In one study (BPD category), there was no male representation. In the rest of the reviewed studies on clinical populations, the male to female ratio was in favor of male participants and ranged from 1.2:1 to 16:1. Aggregating the number of all participants diagnosed with a disorder from one of the above categories across studies reveals a male-to-female ratio of 2.9:1, with 887 male and 306 female participants. In the studies on nonclinical or high-risk populations, in one study [(67); ASD and autistic traits category] all participants were males; in one study [(72)]; Depression measured in a nonclinical or high-risk population category) all participant were females; in six studies the number of male and female participants was even; and in the remaining two studies more females than males participated. When aggregating numbers of all participants in the 10 nonclinical studies, the male:female ratio was 1.16:1. The male:female ratio also differed among categories of clinical condition. As can be seen in **Figure 2**, while studies on psychopathy, ADHD, conduct disorder and callous-unemotional traits, schizophrenia spectrum and psychotic disorders, and ASD and autistic traits relied more on male participants, studies in the categories of depression (depressive traits in a nonclinical or high-risk population), SAD, social anxiety and trait/state anxiety, BPD and risk for Hypomania relied more on female participants.

#### EA Paradigm Used

Most of the studies (22 papers) were based on the emotional story inferring paradigm (or similar); about a third (10 papers) were based on the dyadic interaction paradigm; one study relied on the diary procedure; and one study utilized a similar procedure to that of the daily diary, though slightly modified.

*Limitations mentioned by researchers* were mainly a small sample size, underrepresentation of females in the sample, comorbidity with other conditions, use of medications, and a lack of ethnic diversity among targets.

#### Publishing Year

Although we did not limit the search years, all included papers were published between 1997 and September 2019, with 82% (28/34) published after 2010.

#### Specific Results per Clinical Population

**Table 1** presents the characteristics, main objective, findings, and conclusion of each of the studies included in this review.

In the following section, we review the main findings from the papers included, organized by clinical populations or clinical traits. Categories of clinical populations/traits are presented according to the number of relevant studies found, from the categories with a larger number of studies to those with the fewest. Two exceptions are categories that include papers referring to two different clinical conditions in the same comparative study. In these cases, the category of the clinical condition with the smaller number of studies will follow the category with the larger number. These cases will be explicitly noted when presenting the new category.

### Schizophrenia Spectrum and Psychotic Disorders

Thirty-one percent (eleven papers) of the studies included in the current search focused on EA in the context of schizophrenia and psychotic disorders. In five studies (53, 56, 58, 61, 63), a group of participants with schizophrenia was compared with a nonclinical control group. In Lee et al.’s study (62), a schizophrenia group was compared to both a nonclinical control group and a group of participants diagnosed with bipolar disorder (see below). In de Jong et al.’s study (54), a group of violent participants with a psychotic disorder (a primary diagnosis of schizophrenia or schizoaffective disorder) was compared to a nonviolent psychotic disorder group and to a nonclinical control group. One study (60), which aimed to evaluate psychometric properties of EA and other paradigms to inform possible use in clinical trials, used both a between-group design (schizophrenia group in comparison to nonclinical control group) and a within-subject (test-retest) design in the schizophrenia group. Olbert et al. (59) applied a within-subject design in order to examine the relationship between EA (and other social-cognitive paradigms adapted from social neuroscience) and functionally meaningful outcomes in schizophrenia. Davis et al. (57) assessed whether oxytocin would enhance the effectiveness of a psychosocial intervention—applied both before and after treatment with a double-blind drug administration design. Harenski et al. (55) compared criminal offenders with psychotic disorders to criminal offenders with no history of psychotic disorders and to a nonclinical nonoffenders control group. Within the first group, psychotic offenders with a history of suicide attempts were compared to psychotic offenders without such a history. In all studies but one, the EA tests were based on Zaki et al. (44), where EA assessment is based on the *valence* rating of a target’s emotional states while s/he tells an autobiographical *emotional story*. In Harenski et al. (55), participants watched video clips in which people described autobiographical events, and participants indicated the *content* of emotions the people most likely experienced during the event, and additionally *ranked* the emotions (84).

The number of participants with schizophrenia ranged from 15 (61) to 173 [(59, 60)]—two studies based on the same sample), with the control groups usually similar to or smaller than the schizophrenia group. In three studies (54, 55, 57) all participants were males. In all the other studies there were more male participants than female participants, with a male:female ratio ranging from 1.2:1 (62) to 6.5:1 (61).

A synthesis of findings and conclusions from all studies together indicates *reduced overall EA abilities in people with schizophrenia spectrum disorders in comparison to nonclinical controls*. This is a robust finding that holds cross-culturally (53, 54, 56, 61–63). The EA impairment in schizophrenia is not easily explained by attention or motor deficits (61), and no correlation was found between EA performance and schizophrenia symptoms (63). Also, no significant effects of gender or gender by diagnosis were found on EA (58). However, the valence of the content that targets convey and participants need to infer may be an important variable that moderates results: In one study (58) it was found that the EA impairment in schizophrenia is specific to negative content, while in positive content participants with schizophrenia scored similarly to controls. Interestingly, this difficulty understanding others’ negative affect was associated with lower indices of social support. Harvey et al. (61) found that participants with schizophrenia were more impaired than controls in EA in the context of negative videos compared with positive videos. In Lee et al. (63), both groups showed better accuracy for positive valence; however, participants in the schizophrenia group demonstrated impairment in the positive as well as in the negative valence stimuli.

Findings also indicate that EA is a sensitive measure that captured a group difference between individuals with schizophrenia and controls, even where other tasks (e.g. RMET) did not (58). EA differentiated not only between people with schizophrenia and healthy individuals, but also within a group of patients with a psychotic disorder, EA differentiated between those with and without a violent history (54), and between psychotic offenders with and without past suicide attempts, and nonpsychotic offenders and nonclinical controls (55), where lower EA was associated with a greater likelihood of a past suicide attempt, beyond other risk factors such as depression and substance use. EA was found to be a sensitive differentiating measure in such cases even when other measures (such as ToM, “understanding the other’s mind”) were not (54). In a research project evaluating the psychometric properties of four different social-cognitive paradigms adapted from social neuroscience (basic biological motion, emotion in biological motion, self-referential memory and EA) that were administered to participants with schizophrenia, EA had the broadest external validity (59). The other examined paradigms all had limitations for use in clinical trials, at least without further adaptation (60).

Similar to findings from the general population, associations between EA measures and self-report empathy measures from questionnaires in participants with schizophrenia were found to be weak (53, 56, 63). This might suggest a discrepancy between subjectively experienced empathy and actual empathy performance in a dynamic, interpersonal task. Another possible explanation is that the EA measure captures a certain aspect of empathy, while questionnaires [in these cases: IRI; (32); QCAE, (89)] capture a different aspect. This lack of a significant correlation between EA tasks and empathy self-report questionnaires, alongside the fact that other tasks designed to measure empathy did not always differentiate between participants with schizophrenia and controls (54, 58) may also indicate that at least some aspects of empathy are intact in schizophrenia. It seems that people with schizophrenia do not respond to others’ greater emotional expressivity as much as healthy individuals do (53, 61, 63). Level of expressivity of the targets in all these studies was based on their score on the Berkeley Expressivity Questionnaire [BEQ; (90)], a self-report questionnaire that assesses tendencies to experience and express strong emotions in general (example items: “Whenever I feel positive emotions, people can easily see exactly what I am feeling”; “I sometimes cry during sad movies”; “I’ve learned it is better to suppress my anger than to show it”; “I am an emotionally expressive person”. Van Donkersgoed et al. (53) found that with less expressive targets, participants with schizophrenia and controls had similarly low EA scores, but with more expressive targets, the control group performed better on EA than the patients with schizophrenia. On the neural level, it was found that expressivity elicited activity in specific regions more powerfully in controls than in participants with schizophrenia (61). Lee et al. (63) found that although both schizophrenia and control groups showed greater EA for more expressive targets, this effect was significantly smaller in schizophrenia participants. What seems to improve EA performance in schizophrenia is oxytocin: Participants assigned to oxytocin demonstrated significantly greater improvements than placebo on the measure of EA [but not on other social-cognitive measures; (57)].

Lastly, two studies utilized fMRI (61) and structural MRI (55) scans. Supporting the idea that both mental-state attribution and experience-sharing processes contribute to EA, Harvey et al. (55) found that in healthy controls, EA was associated with increased activity in brain regions typically linked to cognitive effort (i.e., lateral PFC), visual attention (i.e., parietal and occipital cortices), socioemotional processes, including mental-state attribution (i.e., mPFC, precuneus, posterior cingulate), experience sharing (i.e., inferior frontal, inferior parietal), and social context processing (i.e., parahippocampal gyrus). However, in participants with schizophrenia, the pattern of accuracy-related brain activity was relatively sparse (61). Harenski et al. (55) found that offenders with psychotic disorders and suicide attempts demonstrated lower EA and had smaller temporal pole volumes relative to controls, to nonpsychotic offenders and to psychotic offenders without past suicide attempts (this association was significant independent of other risk variables).

### Bipolar Disorder

One of the studies described in the schizophrenia category (62) was a comparative study aiming to determine the relative extent of impairment in social (and nonsocial) cognitive domains in individuals with bipolar disorder compared with schizophrenia patients. EA was thus a part of a battery measuring social cognition within these two groups and in a nonclinical control group. Participants in the bipolar group did not differ from comparison participants on EA, nor in each of the other social-cognitive tasks, whereas schizophrenia patients showed impaired social-cognitive performance compared with both bipolar patients and the control group. Bipolar disorder was found in this study to be associated with less impairment on social relative to nonsocial-cognitive performance, whereas schizophrenia was associated with more impairment on social relative to nonsocial-cognitive performance.

### ASD and Autistic Traits

Twenty-two percent (eight papers) of the studies that were found in the current search focused on EA in the context of ASD or autistic traits. In five of them (64, 68–71), a group of participants with ASD was compared with a nonclinical control group. In one study (66), a group of participants with ASD was compared to both a nonclinical control group and a group of participants diagnosed with ADHD. In two studies (65, 67), autistic traits were assessed in nonclinical samples. In five studies (66, 68–71), participants were mostly high-functioning individuals (sometimes defined as Asperger’s syndrome, or PDD). EA measurement was based on a *dyadic interaction* paradigm, with perceivers asked to infer the *content* of the targets’ mental states (43, 91). In Demurie et al. (66), one of the targets featured in each filmed interaction was diagnosed with ADHD while the other was a typically developed participant. In Ponnet et al. (69), all members of dyads who participated as targets also participated later as participants for measuring EA; each dyad included a participant with ASD and a typically developed participant. Two studies (65, 67) relied on Zaki et al. (44), where EA assessment is based on the *valence* rating of a target’s emotional states while s/he tells an autobiographical *emotional story*. One study focused on empathic *embarrassment* accuracy among individuals with ASD in comparison to the control group, using a similar paradigm (64). One study (67) aimed to test whether variance in social proficiency moderates the effects of *oxytocin* on social-cognitive performance, applying a randomized, double-blind, placebo-controlled design: Participants completed a questionnaire measuring autistic traits [AQ; (92)] and then self-administered intranasal oxytocin or a matching placebo before completing an EA task. EA scores were then compared between the experimental and the control (placebo) group.

The number of participants with ASD ranged from 11 participants (69) to 24 participants (70), with the control groups usually the same size, or slightly larger. Females were generally underrepresented in all six studies with participants with ASD: The number of female participants with ASD ranged from 0 (68) to 5 (71), resulting in an overall male-to-female ratio of around 10:1. In the two studies that were based on a nonclinical sample (65, 67), sample sizes were larger (n = 100; n = 27). In aan het Rot and Hogenelst’s study (65), the male-to-female ratio was 1:1, and in the Bartz et al. study (67), all participants were males.

The synthesis of the findings and conclusions from all the studies together shows that individuals with ASD exhibit a deficit in EA abilities (64, 66, 68–71). More pronounced autistic traits in typically developed individuals were also associated with poorer EA abilities (65, 67). However, this may be true only for individuals who have more autistic traits as well as less trait affective empathy (5). Additionally, this association was found to be moderated by the hormone oxytocin: Bartz et al. (67) showed that oxytocin selectively improved EA for people with more pronounced autistic traits. In this study, participants with less pronounced autistic traits performed better on the EA task in the placebo condition and maintained this performance level in the oxytocin condition, whereas participants with more pronounced autistic traits performed worse in the placebo condition but significantly better in the oxytocin condition, such that in the oxytocin condition, the performance of participants with more and less pronounced autistic traits did not differ. Roeyers et al. (70) found that participants with ASD did not use more time than the control group to complete the EA assessment, while in Ponnet et al. (71), participants with ASD needed more time than the controls to carry out the EA task.

Importantly, the measurement of EA in a naturalistic, ecological paradigm captured the difference between the ASD group and a control group when static mind-reading tasks did not (71). Ponnet et al. (68) found that when participants were presented with two filmed interactions, one more structured than the other, participants with ASD demonstrated better EA abilities on the more structured video than on the less structured one, while no such difference was found in the control group. Thus, the findings from both studies (68, 71) emphasize the role of *structure* in bringing out empathic abilities of individuals with ASD, indicating that they perform better in more structured settings, tasks or situations. Ponnet et al. (69) found that when participants with ASD who had to infer the thoughts and feelings of a target in a videotaped interaction also took part in these prerecorded interactions, they did not differ from a nonclinical control group in their EA scores. The researchers concluded that being in the interaction yields higher EA scores than perceiving a social interaction without participating in it (69). In terms of people with ASD, this may be a result of the opportunity to review a social situation that was previously experienced, hence reflecting practice and learning. It is also possible that the interactive experience itself enhanced EA due to attention, motivation or even bio-behavioral factors, such as oxytocinergic influences. Roeyers et al. (70) noted that although impairments in EA were observed among people with ASD, the underlying *mechanisms* accounting for this remain unexplained. They added that as the advanced EA measure proved to be a valid alternative for the static tests, they believe that future work incorporating the EA paradigm could expand the research on deficient mind reading in ASD.

### Attention Deficit and Hyperactivity Disorder

In one of the studies reviewed above (66), adolescents with ASD (n = 13) were compared to adolescents with ADHD (n = 13) and to a nonclinical control group (n = 18) on EA performance, in a dyadic interaction paradigm. In each dyad, one of two interacting targets was a typically developing adolescent, and the other was diagnosed with ADHD. Thus, participants with ADHD were examined in this study not only as subjects but also as the targets for EA (participants with ASD were examined only as subjects/perceivers). The study results demonstrate the impairment in EA abilities of adolescents with ASD. Participants with ADHD performed as an intermediate category between the ASD and the control group in EA abilities: Their scores did not differ significantly from those of the control group nor from those of the ASD individuals. As targets, participants with ADHD were less accurately understood than the typically developing participants, and their thoughts and feelings seemed to be less easy to read.

### Depression Measured in a Nonclinical or High-Risk Population

In 14% of the papers (five papers), the relationship between EA and depressive traits or states in a nonclinical or high-risk population was examined. Two studies (72, 73) used an EA test based on the valence rating of targets narrating autobiographical stories (44, 65). One of them (73) aimed to examine the effects of reduced brain serotonin on EA, oxytocin and mood in never-depressed individuals with low vs. high risk for major depressive disorder. This study utilized a double-blind cross-over design, with an order of treatment randomized by gender and group (high vs. low risk, 10 males and 10 females in each group, and two treatment conditions). The other study (72) aimed to examine the impact of light therapy on mood and on cognitive empathy in premenstrual women with symptoms indicating a premenstrual disorder (PMS). The sample was characterized by mild depression [assessed using the Quick Inventory of Depressive Symptoms; (93)]. This study utilized a participant-blind between-groups (two treatment groups) design and included 48 females. In both studies, participants’ EA performance was not affected by intervention. aan het Rot et al. (72) found that the therapy improved mood (only in women not using hormonal contraceptives), but found no differential effects of light therapy on EA, even when potential moderators such as valence (positive or negative) of the stimuli, the target’s emotional expressivity, PMS severity, participants’ depression and contraceptive use were taken into account. Similarly, Hogenelst et al. (73) found that the procedure used to model reduced serotonin (acute tryptophan depletion; ATD) did not significantly alter EA in the high-risk group, nor in the control group. In both studies, participants obtained higher EA scores when watching positive stimuli compared to negative stimuli, but without moderating the overall results. To sum, in both studies EA and depression were measured in the context of an intervention aimed to target depression (light therapy, ATD), and in both EA was not affected by the intervention.

The other three studies all used samples of romantic couples [51 couples in Gadassi et al. (74)]; 267 couples in Papp et al. (75); 74 couples in Thomas et al. (76)). These studies measured both EA and depressive symptoms and utilized a dyadic interaction paradigm (Actor–Partner Interdependence Model). All three studies measured EA using a lab procedure where couples are videotaped while interacting (discussing a given topic or an issue of conflict); then they separately review the recording, write the content of their own experienced mental states during the interaction and infer their partner’s mental states. One study (74) additionally utilized the daily diary procedure, measuring both content and valence of the partner’s thoughts and feelings. Thomas et al. (76) examined the predictors of EA and assumed similarity (judgments of how closely linked partner emotions are) in a sample of married couples, in the context of problem-solving discussions, considering depression. They found no association between depression and EA. It is interesting to note, however, that lower levels of depression tended to produce higher levels of assumed similarity. Based on the procedure applied by Thomas et al. (74), Papp et al. (75) tested partners’ EA and assumed similarity in marital conflict interactions, and whether they are moderated by spouses’ levels of depressive symptoms. They found that higher levels of depressive symptoms were associated with reduced EA for negative emotions (among both males and females) and, surprisingly, with increased EA for positive emotions among females. Gadassi et al. (74) aimed to examine gender differences in the association between depressive symptoms and interpersonal perception. In the lab measures, they found that females’ (but not males’) higher levels of depressive symptoms were associated with lower EA. In the daily diary procedure, females’ depressive symptoms were specifically associated with lower levels of EA for negative (but not for positive) feelings, and with lower levels of their partner’s EA for the females’ negative feelings. Males’ depressive symptoms were again unrelated to levels of EA. They concluded that when a woman is depressed, first her own EA is lowered, and second, her partner’s EA when trying to infer her emotional state is also lowered. This pattern was valence-specific and gender-specific. Taken together, findings from these three studies present some inconsistencies regarding the association between EA and depressive symptoms and indicate that the mechanism underlying this potential association may be modified both by valence and by gender.

### SAD and Trait/State Social Anxiety

Three studies examined associations between EA and social anxiety. One study (77) compared 32 participants with a SAD to a nonclinical matched control group. These researchers aimed to compare cognitive empathy and affective empathy in individuals with SAD to that of nonanxious controls. They used an adapted version of an emotional story inferring paradigm (44), adding to the procedure a measure of “empathic congruence.” According to Morrison et al. (77), while perceiver inference of the target’s emotional valence provides a measure of *cognitive empathy*, a measure of *emotional empathy* can be gained by examining the degree of congruence between the target’s self-rating of emotion and the participant’s self-rating of emotion. They found that individuals with SAD did not differ from controls in continuously rating how negative or positive they thought the targets felt (i.e., in EA, cognitive empathy). However, they did differ from controls in their empathic congruence (rating how they themselves felt): For positively valenced (but not for negatively valenced) clips, individuals with SAD exhibited significantly lower empathic congruence.

In the remaining two studies (78, 79), social anxiety was measured in a nonclinical population. Auyeung and Alden (78) examined whether individual differences in social anxiety moderated EA. They randomly assigned 121 participants to an experimental condition designed to increase state anxiety *via* social threat or to a control condition; they then asked the participants to observe videos of target individuals discussing either a socially painful or a nonpainful event. Both targets and participants rated the negative emotions that the targets were feeling while discussing the event. The researchers found that social anxiety was associated with higher EA for others’ negative social emotions (social pain), but only when participants experienced social threat (under the social-threat condition). Simpson et al. (79) tested how people with more anxious-ambivalent attachment orientations [a measure of anxiety in the context of relationships; (94, 95)] react when potential alternative dating partners threaten their relationship. Eighty-two dating couples inferred their partner’s mental states from a videotaped interaction in which they each rated pictures of opposite-sex individuals for attractiveness. EA was operationalized in this study as the degree to which one participant’s inference about the content of each of his or her partner’s thoughts and feelings matched the partner’s actual thoughts and feelings (by independent coders). Highly anxious participants demonstrated higher EA in this relationship-threatening situation. These more anxious participants also showed greater relational instability when they more accurately read their partners’ thoughts and feelings, and their relationships were more likely to have ended 4 months later, measured in a follow-up screening. According to Simpson et al. (79), their findings demonstrate that in relationship-threatening situations, anxious-ambivalent individuals appear to be particularly vulnerable to the negative implications of their partner’s thoughts and feelings.

### Borderline Personality Disorder

Two studies (80, 81) measured EA in the context of BPD. Both utilized a dyadic interaction paradigm. Miano et al. (80) measured EA in 30 romantic couples, with a female partner diagnosed with BPD, in comparison to a control nonclinical group of 37 couples. They aimed to investigate whether females with BPD show *inaccuracy* during a relationship-threatening conversation with their partner (the authors note that motivated inaccuracy is a protective mechanism for couples in healthy relationships during some relationship-threatening situations). Their findings indicate that when facing a relationship-threatening situation, couples in the control group demonstrated inaccuracy, i.e., reduced EA. In contrast, females with BPD tended to increase their EA compared with females in the control group, in a relationship-threatening context. Male partners of BPD females did not differ from males in the control group in the EA pattern.

Flury et al. (81) aimed to explore the “borderline empathy phenomenon,” i.e., the claim, suggested by clinical psychologists, that patients with BPD are unusually accurate at “reading” other people (23–25, 96, 97). The authors used an assessment of EA. They recruited 30 males and 46 females from a larger sample of participants who completed the Borderline Syndrome Index [BSI; (98)], and scored in the upper and lower quartiles, to create a group of individuals at high risk for BPD and a low-risk group. Participants were then assigned to same-sex dyads, each composed of one “borderline” (high-risk) and one “nonborderline” (low-risk) participant, and EA was measured within this dyadic interaction paradigm. Researchers found that the high-risk BPD dyad members displayed better EA than the low-risk BPD dyad members, which seemed to support the borderline empathy phenomenon. However, further analyses [with the Actor–Partner Interdependence Model, APIM; (99–101)] revealed that between those at high risk versus those at low risk, these effects were not a consequence of greater abilities on the part of the BPD participants, but poorer abilities on the part of their partners, meaning that for high-BPD members, EA was harder to predict and more difficult to infer by their partners. The authors emphasize the importance of considering the fact that “high BPD individuals do not have greater empathic ability; they are simply harder to ‘read’.” [(81), p.326]

### Conduct Disorder and Callous-Unemotional Traits

Two studies evaluated EA abilities in the context of conduct disorder in adolescents. In one study, Martin-Key et al. (82) compared male adolescents with Conduct Disorder (CD) and higher versus lower levels of callous-unemotional (CU) traits (n = 37) and a nonclinical control group of male adolescents (n = 40), using an emotional story inferring paradigm. This study employed a modified version of the EA task developed by Zaki et al. (49) in order to draw three measures: the participants’ ability to track changes in the intensity of the target’s emotion, i.e., EA; their ability to recognize the specific emotion displayed by the target after watching the full video clip, i.e., emotion recognition; and the participants’ reported experience of the same emotion as the target, i.e., emotional empathy. They found that relative to controls, participants with CD showed deficits in emotion recognition and emotional empathy (deficits were particularly evident for sadness, fear and disgust), but not in EA. Comparison between the subgroups of high versus low CU traits did not yield any significant differences in EA either.

In the second study, De Ridder et al. (83) assessed EA of male institutionalized adolescents toward staff members, over eight days, in 71 participants with high and low CU traits. Their findings indicate that adolescents with high CU traits perform in the normal range for anger recognition, and they are as accurate as low CU in inferring distress among staff members. The adolescents with high CU traits overestimated the intensity of both anger and distress, in particular during their own misbehavior. The authors suggest that this may reflect overrelying on cognitive empathy ability, instead of their impaired emotional empathy abilities. Thus, the two studies, conducted using two different methods, in two different settings, imply that in the context of conduct disorder, EA as a measure of cognitive EA is intact (when the participant is asked to track the intensity of the target’s emotion). However, accuracy in emotion recognition is impaired, as is the ability to accurately share the affective experience of the target, as a measure of emotional empathy.

### Psychopathy

Surprisingly, only one paper (84) was found in the search to study EA in psychopathy. This study aimed to examine the relationship between psychopathy and cognitive empathy, with a procedure similar to that of the emotional story inferring, using standardized forced-choice response format for both the videotaped targets and the perceivers (and not a continuous rating scale). Findings revealed an inverse association between psychopathy and EA scores, as well as robust group differences between psychopathic and nonpsychopathic male inmates.

### Risk for Hypomania

One study (85) measured the association between EA and high risk for hypomania. The study included 121 participants (57% females) and utilized an emotional story inferring paradigm. The researchers examined how the risk for hypomania contributes to the emotional experiences upon encountering another person’s emotions and EA of that target’s emotions. The risk for hypomania [assessed by The Hypomanic Personality Scale; (102)] was found to be associated with heightened moment-by-moment detection of positive emotions for targets describing positive events, and with overestimating global positive emotion for targets describing negative events. Hypomania risk was also significantly associated with a higher positive emotional experience after viewing a high-intensity negative emotional story video, but not after viewing a low-intensity negative video or high/low-intensity positive video.

### Neurodegenerative Disease

Lastly, one paper (86) investigated the association between EA in patients with neurodegenerative disease and their caregivers’ depressive symptoms. Across two independent studies (n = 172, n = 63), lower EA in neurodegenerative patients was found to be associated with greater depressive symptoms in their caregivers (who were mainly partners). This association was found when accuracy was measured *via* caregiver report or with a dynamic tracking task. Patients’ ability to recognize specific emotions portrayed in photographs or films was not found to be associated with caregivers’ depressive symptoms.

## Discussion

The current review aimed to scope the existing literature on EA in clinical populations. An exhaustive systematic search yielded 34 peer-reviewed papers aiming to measure EA in a clinical population or to assess links between EA and clinical trait or state in a nonclinical or a high-risk population. Overall, the review indicates a growing interest in the EA measure, a dynamic ecological measure that enables greater sensitivity in detecting between-group differences, and more nuanced characterization of empathic functioning.

### An Overview and a Different View of the Main Findings

While ASD and psychopathy are considered to be the two main conditions traditionally associated with empathic dysfunction (11), surprisingly, only one study was found to focus on EA in psychopathy, and two more on conduct disorder in adolescents. The category with the most studies found is schizophrenia (with 31% of the studies). Some of the studies assessed EA in people with a diagnosed clinical condition, while others assessed clinical states or traits in nonclinical or high-risk populations. EA was measured in individuals from clinical groups for various purposes: looking for between-group differences, evaluating interventions and assessing measurements or tools. Accordingly, various designs were used: clinical condition group versus nonclinical control group, randomized or test-retest designs and dyadic designs. Studies also varied in sample sizes and male:female ratios, which will be further discussed.

Almost all studies utilized the emotional story inferring paradigm (or similar), or a dyadic interaction paradigm. These are difficult to compare as they were never used in the same study and were usually used in different contexts or with different populations. For example, the category with the largest number of studies, schizophrenia and psychotic disorders, consists only of studies based on the emotional story inferring paradigm, while all studies focusing on romantic partners used dyadic interactions. This may reflect the tendency of different research groups to use different research paradigms. While there does not seem to be an advantage of one EA paradigm over the other, each has its advantages and limitations. The dyadic paradigm better simulates real-life face-to-face interactions, and it can be used with actual partners expressing emotions from their actual lives together; however, each interaction will end up very different and thus can be difficult to compare. Moreover, this paradigm requires a more demanding coding and scoring process, and it relies on the judgment of raters in assessing the similarity between the target and the perceiver. The emotional story inferring paradigm, on the other hand, is simpler and easier to facilitate as a lab procedure, with the main advantage being the use of the same stimuli for all participants. This can enable a clear separation between the effects of target and perceiver characteristics (as all perceivers see the exact same targets), but it is by nature less ecological. The diary procedure is the most ecological in the sense of having a longer temporal window in which one can examine EA; however, it is suitable mainly for couples, it is the hardest to manipulate and control, and it relies heavily on the participants’ cooperation in their natural environment. Thus, the review does not provide general support for the use of a specific paradigm over the others, but it suggests that scholars should consider the characteristics of each paradigm in light of the research question, the clinical population and the available resources.

Importantly, EA served as a sensitive measure that detected between-group differences even when other paradigms such as emotion detection from still pictures or ToM measures did not (71) in ASD; [(54, 58) in schizophrenia], suggesting that EA, as a complex ecological paradigm, better captures nuanced deficits. However, it is also possible that EA traces a more specific aspect of empathy impairment in these populations that is not captured by the other tasks. A third potential explanation might be that EA tasks are more difficult for these populations due to attention, executive functions, or motor requirements. These observations should be taken into account when planning future studies with clinical populations, and tasks should be made simpler when possible.

An interesting modification was added to some of the reviewed studies, namely, asking participants to report not only on the target’s assumed experience, but also on their own. The authors could then assess not only how accurate participants were in identifying the emotional state of the target, but also how much they themselves shared the target’s affective experience. This addition to the EA paradigm seems to be especially valuable in clinical populations, where deconstructing the multifaceted concept of empathy could contribute to a better understanding of unique clinical profiles. For example, Martin-Key et al. (82) found no impairment in the classic EA measure in study participants with conduct disorder, but found a difference in levels of shared experience, which they referred to as emotional empathy. Similarly, Morrison et al. (77), who studied individuals with SAD, measured both EA and *empathic congruence*, comparing the subjects’ continuous rating of their own emotion to the targets’ ratings. They, too, found no impairment in EA but did find significantly lower empathic congruence in a group of individuals with SAD compared to a nonclinical control group.

Overall, reduced EA was found in schizophrenia, ASD, and psychopathy when compared with nonclinical control groups, and also when compared to individuals with bipolar disorder (in schizophrenia) or ADHD (in ASD). In the context of depression, lower EA was found in the context of negative emotional content (for both males and females), and in higher levels of depressive symptoms in females, but not in males (74). However, other findings indicated no correlation between depression and EA (76)—and even, in certain contexts, an association between higher depressive symptoms and higher EA (75). Negative emotional content conveyed by the target was also associated with lower EA in schizophrenia (58, 61), and this pattern may be true for healthy individuals as well (63). Thus, it may be concluded that negative emotional content is harder to infer accurately, and that gender and clinical condition are among the variables that moderate this specific difficulty. Other variables found to be associated with lower EA were reduced social support (58), the subjects’ history of violence (54), smaller temporal pole volumes and past suicide attempts (55). Note, however, that all the above were inferred from the schizophrenia cohorts and may not apply to the general population. Importantly, reduced EA was also evident when participants were asked to infer the thoughts and feelings of targets with ADHD (66) or with BPD (81), meaning that individuals with these clinical conditions were “harder to read.” This pattern is demonstrated specifically for depressive tendencies of females within marital relationships, where depression in females was found to be associated with reduced EA in both the females and their partners (74). Interestingly, this was not the case for targets with ASD, who did not differ from TD targets in their “readability” [i.e., did not yield lower EA scores in perceivers; (69)].

A clinical condition that has been hypothesized to be associated with enhanced EA is BPD (23–25, 96, 97). Miano et al. (80) found support for this hypothesis only in females in a romantic relationship setting, and specifically in a relationship-threatening situation. However, Flury et al. (81) remind us that when comparing between a clinical and a nonclinical group within a dyadic setting, EA scores are relative and are not independent. If one group gets a higher score than the other, it may imply better EA, or it may hint at more difficulty inferring from the clinical group as targets. Indeed, after utilizing an Actor–Partner Interdependence Model, that was the authors’ conclusion.

A clinical population that does seem to exhibit enhanced EA is SAD, specifically under the experience of social threat (78) or when in a relationship-threatening situation (79). These findings imply better performance under social threat, which may be explained by higher arousal, greater attention or higher motivation in such situations. It is important to note that higher EA may not always be an advantage. For example, Miano et al. (80) claim that the pattern of empathic *inaccuracy* that they found among participants with a low risk for BPD is an adaptive skill in a relationship-threatening situation.

EA performance was improved by oxytocin in schizophrenia (57) and in people with more pronounced autistic traits (67). Feldman et al. (103) found that face-to-face synchronized parent-child interaction had the effect of normalizing oxytocin level in children with ASD, and keeping it high during social contact. This role of social interaction in elevating oxytocin levels in individuals with ASD, alongside the findings on the association between oxytocin and improved EA performance, may relate to the next variable that was found to be associated with better EA functioning in ASD: participation.

In ASD, it was found that participating in the same dyadic interaction that they later had to rate contributed to better EA, compared to inferring from passive observation (69). This could indicate an advantage for the participation itself (over observation), an advantage for learning and rehearsing, or both. Higher arousal, immediate feedback, attention and motivation may also explain this effect. Another variable that was found to be associated with improvement in EA abilities among participants with ASD was the extent to which the situation was more or less structured, i.e., how clear and predictable the social interaction was. ASD participants specifically benefited from a structured versus unstructured situation (68). Thus, in order to better characterize EA in ASD, it is desirable to simulate the complex, dynamic and unstructured daily social interactions, while in planning intervention programs it may be of great value to take into account the potential importance of participation and the role of a structured social situation in encouraging the EA abilities of people with ASD.

Given the dyadic nature of empathy, both the target and the perceiver contribute to EA. The perceiver’s ability to accurately infer the target’s thoughts and feelings depends not only on his/her states and traits but also on the various characteristics of the target, such as expressivity and motivation. Though some studies referred to such “target effects” on EA [e.g., (53, 63, 66, 75)], much of the reviewed literature emphasized the perceiver’s side. Specifically, many studies were designed to investigate whether a clinical condition affects the perceiver’s EA performance [e.g., (54–56, 58, 64, 65)]. Another interesting question that is highly relevant on both a social and a clinical level is how a clinical condition of a target affects the way perceivers understand the target’s emotional state. Moreover, a target–perceiver interaction effect must also be considered. Such questions relate to the growing literature on “the double empathy problem,” which stresses that it’s not only autistic people who struggle with empathy—neuro-typical people also struggle to understand the minds of autistic individuals and empathize with them (104, 105). Such ideas challenge the traditional framing of autism as entailing empathic dysfunction. EA measures can be helpful in investigating this dyadic nature of empathy, as they rely on both the target and the perceiver’s reports. Such directions are suggested by the findings of Flury et al. (81) on BPD patients’ “readability,” and of Demurie et in the context of ADHD, and may be of great value in the study of other clinical conditions as well.

We suggest that these variables, discussed in the context of either impairing or enhancing EA, can be further classified as *subject variables* (e.g., the clinical condition, clinical profile, biological characteristics, previous experiences, participation, social support), *target variables* (such as expressivity, content conveyed, clinical condition and specific profile), and *situational variables* (e.g., structured vs. unstructured, threatening, familiar). Within this framework, wherein the subject, the target or the situation can influence EA results, it may be valuable to consider additional variables in future research on empathy in clinical populations. One such variable that was barely directly addressed in the reviewed papers, yet was very pronounced in the process of synthesizing the findings, is gender.

The current review reveals a general underrepresentation of female participants in studies on clinical populations, and a slight underrepresentation for males in studies aiming to evaluate clinical traits or states in nonclinical or high-risk samples. This finding may reflect either a trend in research questions and aims, recruitment challenges (sometimes due to male:female ratio in a specific condition) or both. One consequence of this trend is that while in nonclinical studies gender differences can be (and sometimes are) examined, in studies based on clinical samples, the associations between EA, gender and clinical condition are hardly addressed. For example, without considering the male:female ratio of participants in each study, one might conclude that EA is impaired in ASD, schizophrenia, psychopathy and conduct disorder, and that EA is intact in bipolar disorder, enhanced to some extent in borderline disorder, and that in SAD the dysfunction is due to a lack of protective inaccuracy. But a closer look at the gender of participants in each category reveals that while studies in ASD, schizophrenia, psychopathy and conduct disorder were done mostly on male participants, research on BPD and SAD relied more on female participants. To date, in most studies on clinical populations, the sample size is not large enough to address this question, with the recruitment of clinical participants and specifically females constituting one of the main challenges limiting the studies, as researchers themselves often note (57, 61, 64, 67).

We believe that findings regarding gender, clinical phenotype and EA interactions may have important clinical implications. For example, Gadassi et al. (74) state that according to their findings, when females are depressed, their romantic relationship suffers doubly: first, because their own EA is lower, and second, because their partner’s EA is also lower. In contrast, when males are depressed, neither their own nor their partner’s levels of EA change. In the field of autism research, for example, there is a growing understanding that the male:female ratio might be different than previously assumed (106–108). New research indicates that females with autism are underdiagnosed and understudied, due to lack of knowledge on the ASD female phenotype, and perhaps to the “camouflage effect” [an hypothesis that females with ASD are better at camouflaging their social deficits; (109)]. Along these lines, we encourage future studies to take gender into account, and call for a deeper investigation of a potential clinical profile, EA and gender interaction.

Lastly, we want to draw attention to a group of studies focusing on EA in the context of violent or aggressive behavior in intimate relationships. These studies did not appear in our systematic search based on the chosen search-words but were brought to our attention by a reviewer, and we agree that they are of clear relevance to this review, as aggressive behavior may relate to various clinical conditions (16). For example, Schweinle et al. (110) investigated whether husbands’ wife-directed aggression is related to unusual accuracy (hypersensitivity), or to a bias to infer criticism or rejection inappropriately when they infer women’s thoughts and feelings. They used a procedure similar to the dyadic interaction paradigm to assess EA: videotapes depicting female clients participating in a simulated individual psychotherapy session with the same male therapist, focusing on intimate relationships. Each client watched her filmed therapy session and wrote down her thoughts and feelings through the session [originally developed by Marangoni et al. (91)]. The study’s participants (all males) were asked to infer the client’s thoughts and feelings while watching the videos. Then independent raters rated the similarity between the client’s self-report and the participant’s inferences of her thoughts and feelings. The results revealed that the greater the husbands’ bias to overattribute criticism and rejection to the thoughts and feelings of women they had never met, the more they reported behaving in a verbally aggressive way toward their own wives. The men’s overattribution bias, i.e., inaccurately inferring that women’s thoughts and feelings are critical or rejecting of their male partners, was related not only to aggression against their wives but to the men’s insecure attachment style [see also Clements et al. (111)]. An interesting future research direction may be to study both the violent individuals’ EA *and* their clinical profiles. Such an investigation can also examine whether the association between the aggression and the EA profile is unique to the intimate relationship context, or if it reflects a more pervasive personality characteristic.

### Strengths and Limitations of the Current Review, and Suggestions for Future Research

To ensure a broad search of the literature, the search strategy included PsycNET and PubMed, as well as the snowball technique (also using Google Scholar search engine), and an updated search was performed in September 2019. This review may not have identified all published papers on EA in clinical populations despite attempts to be as comprehensive as possible. Thus, the main limitation of this study is the possibility that the review may have missed some relevant papers, as the search included many words and terms, and it was spread over many clinical populations and research fields. We did not review unpublished studies such as dissertations, which may have contributed additional knowledge. Exclusion of the gray literature from the search and exclusion of studies published in a language other than English has probably left some valuable information outside the scope of this review.

As our aim was to present an overview of the existing literature on EA in clinical populations, we included all eligible peer-reviewed studies, regardless of methodological quality. Future research should address the methodological issues and aim for a meta-analysis of suitable and well-designed studies. This may be of great value in light of the small sample sizes typical of studies on clinical populations.

It seems that the study of EA in clinical populations could benefit from a modified measure that can capture both EA and empathic congruence (77), or accuracy in sharing the affective experience (82). On the other hand, it is reasonable to assume that clinical populations are even more susceptible than nonclinical populations to the length of a task/fatigue effects, and both those considerations must be taken into account when planning a study evaluating EA in a clinical sample.

Research on EA in clinical populations has added to the accumulating knowledge on the price one pays for not accurately understanding others’ affective and mental states. Another interesting and potentially important question refers to the experience of the targets when they are not being understood. We have learned that low EA is associated with depression in the *partners* of the clinical patients with low EA (74, 75, 102). Thus, people who are not accurately understood on a daily basis suffer from the other, less-studied side of the EA model. Therefore, future studies could benefit from not only examining EA in a relevant clinical population, but exploring the effects of EA difficulties on spouses, family members and other social partners as well. It would also be relevant to examine individuals with clinical conditions not just as subjects or perceivers but also as *targets* of EA, i.e., to study not only how accurate individuals with social deficits are at understanding the other and sharing their emotions, but how accurately they are being understood by others, and possible associations with well-being.

To the best of our knowledge, this is the first review of the existing literature on EA in clinical conditions, states and traits. It reveals a growing interest in using these measures to deepen our understanding of clinical profiles, and it indicates that EA assessments have the potential to capture unique and subtle characteristics of empathic function and dysfunction. It also points to the paucity of existing studies on EA in the context of most clinical conditions. Due to the variance between and within clinical populations, and the variety of research aims, designs and methods across existing studies, it is difficult to draw robust meta-analytic conclusions regarding the nature of EA in clinical populations. A promising future research direction would be to integrate the cumulative knowledge on EA in the general (nonclinical) population with emerging data from clinical populations. For example, in the studies reviewed here, anxiety was found to be associated with enhanced EA in a relationship-threatening situation ((79); see also 93 for similar results with BPD). Ickes & Simpson (112) refer to motivational inaccuracy as protective in intimate relationship under certain circumstances of threat to the relationship, and it seems that anxiety and BPD are associated (perhaps only in females) with not applying this protective behavior. An alternative explanation is that enhanced EA is associated with enhanced alertness, sensitivity or arousal, which may characterize BPD patients as well as anxious individuals and individuals under threat [support for such an interpretation also can be found in Auyeung and Alden (78), and Devlin et al. (85)]. This interpretation may account for Ponnet et al.’s finding in which participants with ASD did not differ from a control group in their EA scores after participating in an interaction with the target (69). It may be that real face-to-face interactions cause increased alertness and arousal, and these facilitated EA. More research is needed in order to disentangle the role of personality traits and emotional states in EA in both clinical and nonclinical populations.

In summary, EA is an important measure, paradigm and concept in empathy research in the context of clinical populations. Though some limitations to the use of specific tools for measuring EA in clinical populations need to be considered, it seems that EA paradigms are promising for measuring outcomes and discriminating clinical from nonclinical populations, and subgroups within clinical conditions, even when other paradigms fail to do so. It may be that with further advances in research, EA paradigms could be used as a screening tool, and maybe even in training and practicing empathic abilities. In future research on EA in clinical populations, we suggest addressing understudied populations, such as psychopathy. Subject, target and situational variables should be considered, with special attention to gender differences (and similarities), the association between EA abilities and adaptive functioning, and the study of individuals with clinical conditions as targets of EA. These avenues of investigation may promote a better understanding of the nature of EA, of specific clinical profiles and of social attitudes toward people with clinical conditions.

## Author Contributions

Both YR and AP equally contributed to the review.

## Funding

This work was supported by an Azrieli Fellowship from the Azrieli Foundation to AP.

## Conflict of Interest

The authors declare that the research was conducted in the absence of any commercial or financial relationships that could be construed as a potential conflict of interest.
